# Haemodynamic changes in cirrhosis following terlipressin and induction of sepsis—a preclinical study using caval subtraction phase-contrast and cardiac MRI

**DOI:** 10.1007/s00330-020-07259-w

**Published:** 2020-10-12

**Authors:** Manil D. Chouhan, Stuart A. Taylor, Alan Bainbridge, Simon Walker-Samuel, Nathan Davies, Steve Halligan, Mark F. Lythgoe, Rajeshwar P. Mookerjee

**Affiliations:** 1 Centre for Medical Imaging, Division of Medicine, UCL, University College London (UCL), London, UK; 2 Department of Medical Physics, University College London Hospitals NHS Trust, London, UK; 3 Centre for Advanced Biomedical Imaging, Division of Medicine, UCL, University College London (UCL), London, UK; 4 Institute for Liver and Digestive Health, Division of Medicine, UCL, Royal Free Hospital, University College London (UCL), NW3 2PF, London, UK

**Keywords:** Liver, Haemodynamics, Sepsis, Terlipressin, Liver Cirrhosis

## Abstract

**Objectives:**

Effects of liver disease on portal venous (PV), hepatic arterial (HA), total liver blood flow (TLBF), and cardiac function are poorly understood. Terlipressin modulates PV flow but effects on HA, TLBF, and sepsis/acute-on-chronic liver failure (ACLF)-induced haemodynamic changes are poorly characterised. In this study, we investigated the effects of terlipressin and sepsis/ACLF on hepatic haemodynamics and cardiac function in a rodent cirrhosis model using caval subtraction phase-contrast (PC) MRI and cardiac cine MRI.

**Methods:**

Sprague-Dawley rats (*n* = 18 bile duct–ligated (BDL), *n* = 16 sham surgery controls) underwent caval subtraction PCMRI to estimate TLBF and HA flow and short-axis cardiac cine MRI for systolic function at baseline, following terlipressin and lipopolysaccharide (LPS) infusion, to model ACLF.

**Results:**

All baseline hepatic haemodynamic/cardiac systolic function parameters (except heart rate and LV mass) were significantly different in BDL rats. Following terlipressin, baseline PV flow (sham 181.4 ± 12.1 ml/min/100 g; BDL 68.5 ± 10.1 ml/min/100 g) reduced (sham − 90.3 ± 11.1 ml/min/100 g, *p* < 0.0001; BDL − 31.0 ± 8.0 ml/min/100 g, *p* = 0.02), sham baseline HA flow (33.0 ± 11.3 ml/min/100 g) increased (+ 92.8 ± 21.3 ml/min/100 g, *p* = 0.0003), but BDL baseline HA flow (83.8 ml/min/100 g) decreased (− 34.4 ± 7.5 ml/min/100 g, *p* = 0.11). Sham baseline TLBF (214.3 ± 16.7 ml/min/100 g) was maintained (+ 2.5 ± 14.0 ml/min/100 g, *p* > 0.99) but BDL baseline TLBF (152.3 ± 18.7 ml/min/100 g) declined (− 65.5 ± 8.5 ml/min/100 g, *p* = 0.0004). Following LPS, there were significant differences between cohort and change in HA fraction (*p* = 0.03) and TLBF (*p* = 0.01) with BDL baseline HA fraction (46.2 ± 4.6%) reducing (− 20.9 ± 7.5%, *p* = 0.03) but sham baseline HA fraction (38.2 ± 2.0%) remaining unchanged (+ 2.9 ± 6.1%, *p* > 0.99). Animal cohort and change in systolic function interactions were significant only for heart rate (*p* = 0.01) and end-diastolic volume (*p* = 0.03).

**Conclusions:**

Caval subtraction PCMRI and cardiac MRI in a rodent model of cirrhosis demonstrate significant baseline hepatic haemodynamic/cardiac differences, failure of the HA buffer response post-terlipressin and an altered HA fraction response in sepsis, informing potential translation to ACLF patients.

**Key Points:**

Caval subtraction phase-contrast and cardiac MRI demonstrate:

*• Significant differences between cirrhotic/non-cirrhotic rodent hepatic blood flow and cardiac systolic function at baseline.*

*• Failure of the hepatic arterial buffer response in cirrhotic rodents in response to terlipressin.*

*• Reductions in hepatic arterial flow fraction in the setting of acute-on-chronic liver failure.*

## Introduction

Complications of liver cirrhosis such as portal hypertension are underpinned by major changes in the dual portal venous (PV) and hepatic arterial (HA) blood supply to the liver[1]. In health, total liver blood flow (TLBF) is regulated closely so that reduction of PV flow triggers increased HA flow—the hepatic arterial buffer response [2]. This response is thought to be impaired in liver disease, but remains poorly understood because references tests are invasive and are confounded by vessel instrumentation, so that they are unfeasible in disease or impractical in smaller animal models [3].

Vasoactive drugs may be used to manage vascular complications of liver disease but are controversial. Terlipressin is a long-acting vasopressin analogue, used to manage acute variceal haemorrhage [4] and type 1 hepatorenal syndrome [5]. Action on V1a receptors in the splanchnic vascular bed reduces PV blood flow [6], but arterial vasoconstrictive properties are associated with serious adverse effects [7]. Compensatory increases in HA flow have been demonstrated in naïve porcine studies [8], but the effects of terlipressin on TLBF and HA flow in the context of chronic liver disease have not been reported previously.

Acute-on-chronic liver failure (ACLF) refers to the development of severely deranged hepatic function and extrahepatic organ failure on a background of chronic liver disease [9]. It is commonly triggered by sepsis and is associated with high short-term mortality comparable with acute liver failure [10]. ACLF onset is associated with portal hypertension–related gut bacterial translocation, which in combination with the systemic inflammatory response exacerbates pre-existing cirrhotic systemic circulatory/cardiac dysfunction [11, 12]. Despite this, reports of the effects of ACLF on hepatic and cardiac haemodynamics are limited [13, 14]. Studies with robust non-invasive protocols to evaluate complications of chronic liver disease and to guide development of new therapeutic haemodynamic modulatory agents are required.

Caval subtraction phase-contrast magnetic resonance imaging (PCMRI) is used to estimate TLBF and HA flow [15]. It is based on two-dimensional (2D) PCMRI (a routinely available sequence to measure large vessel bulk flow) and has been validated invasively in rodents, is reproducible in normal human volunteers and translated into patients with chronic liver disease, where caval subtraction HA fraction has been correlated with portal hypertension severity [15, 16]. Cardiac cine MRI with short-axis views through the left ventricle (LV) is an established non-invasive method to measure stroke volume that in conjunction with heart rate can characterise LV systolic function [17].

In this study, we used caval subtraction PCMRI and cardiac cine MRI in a rodent model of cirrhosis to (a) characterise baseline hepatic haemodynamic and cardiac differences, (b) investigate the hepatic arterial buffer response to terlipressin and (c) investigate the hepatic haemodynamic and cardiac effects of ACLF.

## Materials and methods

### Subjects and preparation

All experiments were conducted according to the Home Office guidelines under the UK Animals in Scientific Procedures Act (1986) after approval from the Animal Care Ethical Committee of University College London. Experiments were performed on healthy male Sprague-Dawley rats (Charles River UK, Margate, England, 250–300 g) with normal liver function.

We investigated 34 healthy animals subject to either sham laparotomy (*n* = 16) or bile duct ligation (BDL, *n* = 18) as described previously [18]. Once recovered, animals were maintained for 4 to 5 weeks to allow secondary biliary cirrhosis to develop in the BDL cohort.

Prior to scanning, anaesthesia was induced with isoflurane gas and a fine bore polyethylene line (Portex, Smiths Medical) was cited in the jugular vein. A rectal probe (SA Instruments) monitored core body temperature, maintained between 36 to 38 °C using circulating warm water pipes and warm air. A triple-electrode single-lead system (SA Instruments) was used for cardiac monitoring. All procedures were performed by the study coordinator (M.C., a radiology research fellow qualified in animal handling).

### Scanning protocol

#### Caval subtraction PCMRI

Scanning was performed on a 9.4-T MRI unit (Agilent Technologies), with sequence parameters as listed in Table 1. Gradient-echo anatomical imaging was used to plan 2D cine PCMRI studies orthogonal to the inferior vena cava and PV. Measurements used prospective cardiac and respiratory gating, a 192 × 192 acquisition matrix, 10° flip angle and 2 mm slice thickness. Velocity encoding (*V*_*enc*_) was 33 cm/s for the PV and infra-hepatic supra-renal inferior vena cava, and 66 cm/s in the supra-hepatic sub-cardiac inferior vena cava. Acquisition time for 12 to 15 phases through the cardiac cycle was less than 10 min. Phase maps obtained in opposite flow-encoding directions were subtracted to correct for background phase errors and final phase-velocity maps were analysed using manually positioned regions-of-interest on each vessel for each frame of the cardiac cycle, using in-house developed MATLAB code (MathWorks).

**Table 1:** Sequence parameters

	Anatomical images (gradient-echo MRI)	PCMRI	Cardiac cine MRI
TR/TE (ms)	8.2/5.6	10/1.2	7.5/1.2
Flip angle (°)	20	10	15
Matrix size (pixels)	128 × 128	192 × 192	128 × 64
Field-of-view (mm)	80 × 80	40 × 40	40 × 40
Spatial resolution (mm^2^)	0.625 × 0.625	0.208 × 0.208	0.313 × 0.625
Slice thickness (mm)	2	2	1
Slice gap (mm)	4.5	-	0
Cardiac cycle phases	-	12-15	≥ 20

As described previously [15], hepatic vascular outflow can be estimated by subtracting infra-hepatic supra-renal inferior vena caval flow from supra-hepatic sub-cardiac inferior vena caval flow. The total volume of blood flowing into and out of the liver is assumed to be constant over the cardiac cycle. HA flow is estimated by subtracting direct 2D PCMRI measurements of PV flow from caval subtraction PCMRI-estimated TLBF. Estimated TLBF, PV and estimated HA flow measurements were all normalised to explanted liver weight (ml/min/100 g). HA fraction (HA flow as a percentage of estimated TLBF) was also calculated.

#### Cardiac cine MRI

As described previously [19], cardiac and respiratory-gated gradient-echo coronal images of the thorax, followed by left ventricular (LV) long-axis images of the heart, were obtained for LV short-axis view planning. Contiguous 2 mm LV short-axis slices (median 6, range 5-8 slices) were obtained from the LV apex to the mitral valve orifice using prospective cardiac and respiratory-gated spoiled gradient-echo imaging with a 128 × 64 acquisition matrix, 40 × 40 mm^2^ field-of-view and 15° flip angle. Acquisition time for at least 20 frames through the cardiac cycle with full LV coverage was typically just under 12 min. Data were analysed using Segment (Medviso), with automatic endocardial and epicardial segmentation and frame-by-frame manual review and correction where appropriate. Stroke volume (mls) was calculated as the difference between LV end-diastolic and end-systolic volumes, cardiac output (ml/min) was calculated as the product of stroke volume and heart rate (bpm), LV ejection fraction (%) was calculated as percentage stroke volume of LV end-diastolic volume, LV mass (g) was calculated as the product of LV myocardial volume (the difference between LV epicardial and endocardial volume at end diastole) and 1.05 g/cm^3^ (the density of myocardial tissue) [20], cardiac index (ml/min/kg) and LV mass index (g/kg) were calculated by dividing the cardiac output and LV mass respectively by body weight.

### Study design and intervention protocol

**Fig. 1:**
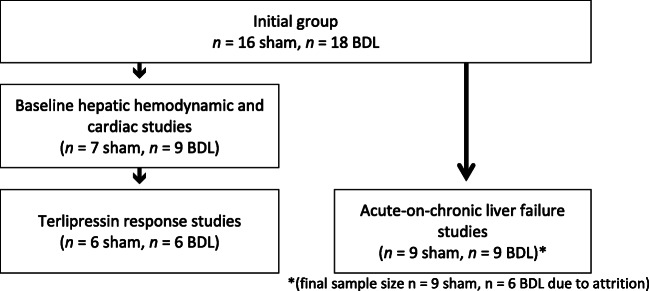
Study cohort

Animals were divided into two study groups (Fig. 1).

#### Study group 1: Baseline hepatic haemodynamic, cardiac and terlipressin response studies

Baseline caval subtraction PCMRI and cardiac cine MRI studies were undertaken in this cohort. To minimise the number of animals used, a sub-cohort of the same animals from this study were used for terlipressin response studies.

Initial feasibility studies in four animals (not presented in this study) were undertaken to determine the drug dosage necessary for a sustained reduction in PV flow. After baseline caval subtraction PCMRI and cardiac cine MRI studies, intravenous terlipressin acetate (Glypressin, Ferring Pharmaceuticals) 100 μg/kg bolus was administered via the jugular line, followed by an infusion of 10 μg/kg/min for 40 min. Ten minutes after the bolus dose, post-terlipressin caval subtraction PCMRI studies were undertaken.

#### Study group 2: Acute-on-chronic liver failure studies

Inflammatory stress generated by lipopolysaccharide (LPS) challenge was used to model ACLF. Dosage was based on prior studies [21]. Continuous intravenous fluid resuscitation with normal saline was administered via the jugular line at a rate of 8 ml/kg/hour. After baseline caval subtraction PCMRI and cardiac cine MRI studies, 0.3 mg/kg LPS (*E. coli* LPS, Sigma-Aldrich) was infused over 60 min, followed by resumption of normal saline fluid resuscitation. Ten minutes after the LPS infusion had been given, post-LPS caval subtraction PCMRI and cardiac cine MRI studies were undertaken.

### Power calculations and statistical analysis

Acute-on-chronic liver failure studies were prioritised to calculate sample size. Calculations for repeated measures two-way analysis of variance (ANOVA) (sham/BDL vs baseline/post-LPS) with differences in TLBF were used as the endpoint variable. Powering at 90% with a 5% significance level with a view to detecting a TLBF response difference of at least 20% between sham/BDL rats (informed from clinical ACLF studies demonstrating larger differences[13]), a sample of *n* = 6 rats per animal type was advised. Assuming an attrition rate of 30% (based on previous experience of LPS in BDL rats), the final projected sample size was *n* = 9 for each group.

Statistical analyses were undertaken using Prism (GraphPad, version 6.01). Data normality was confirmed with Kolmogorov-Smirnov testing. Baseline hepatic haemodynamic and cardiac parameters were compared in sham and BDL groups using unpaired Student *t* tests, with Welch’s correction to account for different standard deviations. Paired hepatic haemodynamic (terlipressin, LPS) and cardiac (LPS) measurement changes in sham and BDL groups were evaluated using repeated measures two-way ANOVA, citing the *F* (between groups degrees of freedom, within-groups degrees of freedom) statistic and with post hoc Tukey tests using Bonferroni’s correction. Data were expressed as means ± standard errors, with significance at the 5% threshold.

## Results

### Cohort features

Four weeks post-surgery, body weight was significantly lower in BDL (428 ± 10 g) compared with sham-operated rats (470 ± 5 g, *p* = 0.0011), but BDL wet liver mass was higher (33 ± 2 g vs 15 ± 1 g, *p* < 0.0001). From the original groups, sample sizes varied for subsequent studies, summarised in Fig. 1.

### Baseline hepatic haemodynamic and cardiac differences

**Fig. 2:**
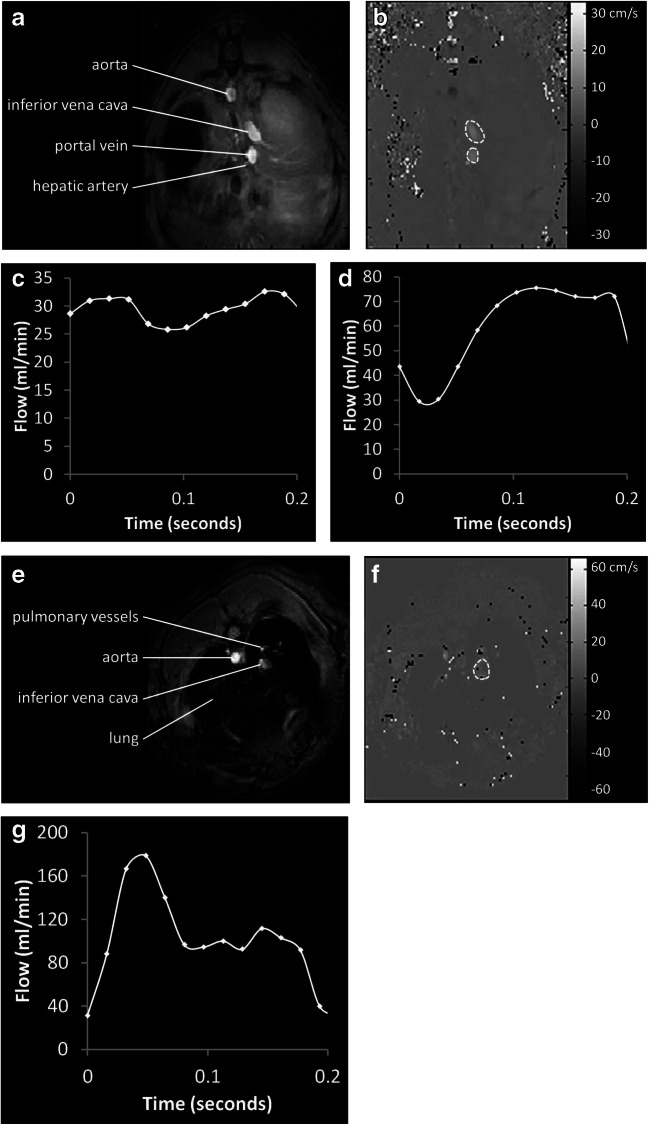
Caval subtraction PCMRI data from a sham-operated rat. Magnitude, matched phase-contrast velocity maps with segmented vessels shown as dashed white ROIs and corresponding flow curves through the cardiac cycle for the portal vein (**a**, **b**, **c**), proximal IVC (**a**, **b**, **d**) and distal IVC (**e**, **f**, **g**). In this example, measured PV flow was 29.9 ml/min, caval subtraction TLBF was 41.8 ml/min and HA flow was 11.9 ml/min

**Fig. 3:**
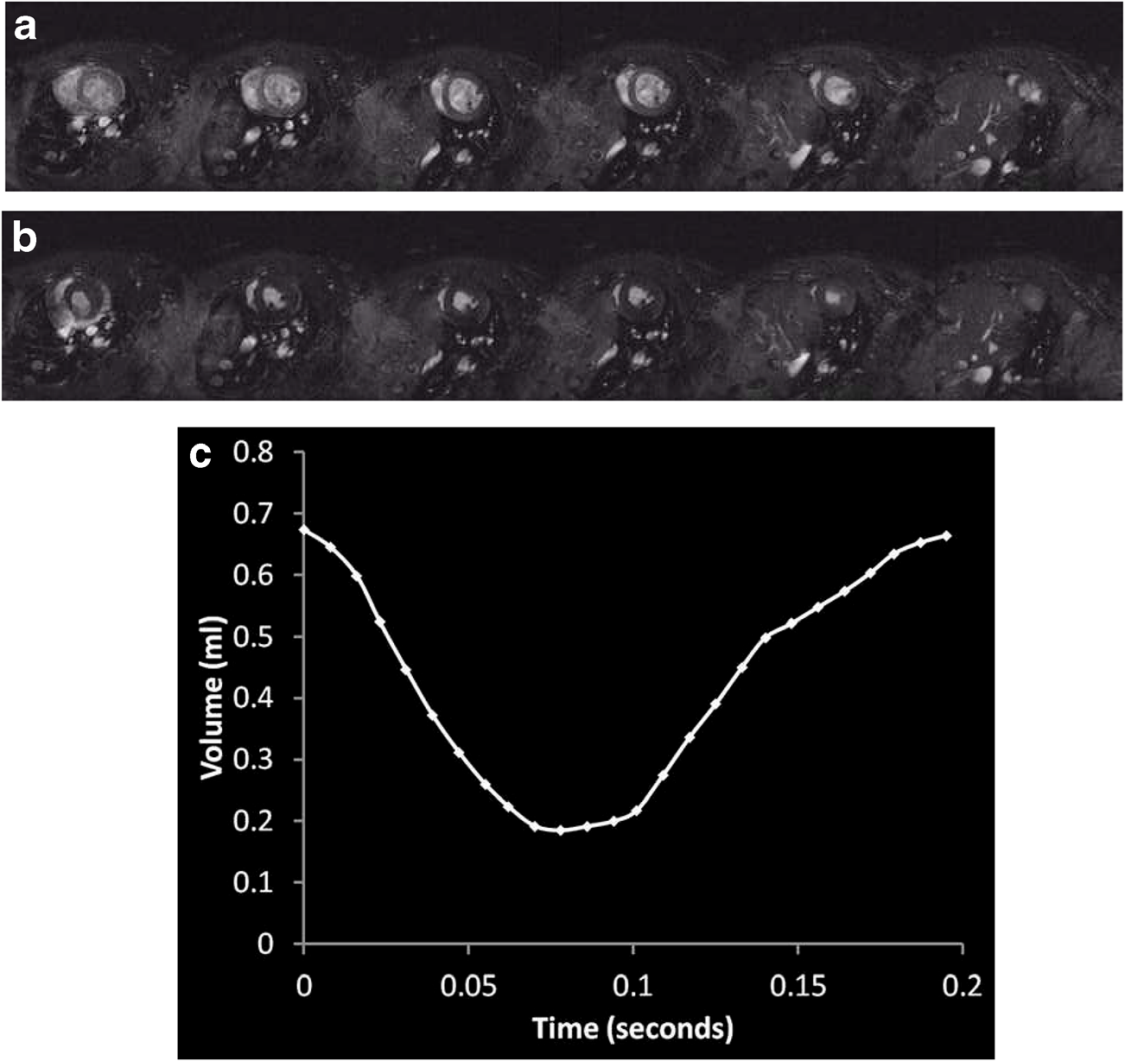
Cardiac cine MRI images from a sham-operated rat, with 6 × 2 mm short-axis slices shown through the left ventricle at (**a**) end diastole and (**b**) end systole. Endocardial segmentation through all phases of the cardiac cycle was used to generate (**c**) LV volume-time curves

Results are summarised in Table 2 and an examples of a caval subtraction PCMRI and cardiac cine MRI studies are shown in Figs. 2 and 3. Baseline mean PV flow (sham 181.4 ± 12.1 vs BDL 68.5 ± 10.1 ml/min/100 g, *p* < 0.0001) and TLBF (sham 214.3 ± 16.7 vs BDL 152.3 ± 18.7 ml/min/100 g, *p* = 0.03) were significantly lower in BDL compared with sham-operated animals. Conversely, HA flow (sham 33.0 ± 11.3 vs BDL 83.3 ± 19.1 ml/min/100 g, *p* = 0.04) and HA fraction (sham 14.4 ± 4.4 vs BDL 51.5 ± 6.8%, *p* = 0.0005) were significantly higher in BDL versus sham-operated animals.

**Table 2:** Baseline sham and BDL hepatic haemodynamic and cardiac systolic function parameters

	Sham	BDL	*p* value
Cohort features			
Body weight (g)	470 ± 5	428 ± 10	0.0011*
Wet liver weight (g)	15 ± 1	33 ± 2	< 0.0001***
Hepatic haemodynamic parameters			
PV flow (ml/min/100 g)	181.4 ± 12.1	68.5 ± 10.1	< 0.0001***
HA flow (ml/min/100 g)	33.0 ± 11.3	83.8 ± 19.1	0.0404*
HA fraction (%)	14.4 ± 4.4	51.5 ± 6.8	0.0005**
TLBF (ml/min/100 g)	214.3 ± 16.7	152.3 ± 18.7	0.0266*
Cardiac systolic function parameters			
Heart rate (bpm)	333 ± 8	341 ± 9	0.5264
End-diastolic volume (ml)	0.66 ± 0.02	0.77 ± 0.03	0.0188*
End-systolic volume (ml)	0.25 ± 0.02	0.20 ± 0.01	0.0732
Stroke volume (ml)	0.42 ± 0.02	0.57 ± 0.04	0.0059*
Cardiac output (ml/min)	140.0 ± 8.0	195.5 ± 15.2	0.0074*
LV ejection fraction (%)	63.2 ± 2.4	73.7 ± 2.8	0.0127*
Cardiac index (ml/min/kg)	291.5 ± 13.3	456.1 ± 33.4	0.0009**
LV mass (g)	0.90 ± 0.06	0.98 ± 0.06	0.3367
LV mass index (g/kg)	1.87 ± 0.13	2.30 ± 0.13	0.0343*

Unpaired sham vs BDL Student *t* tests

*p < 0.05; **p < 0.001; ***p < 0.0001

**Fig. 4:**
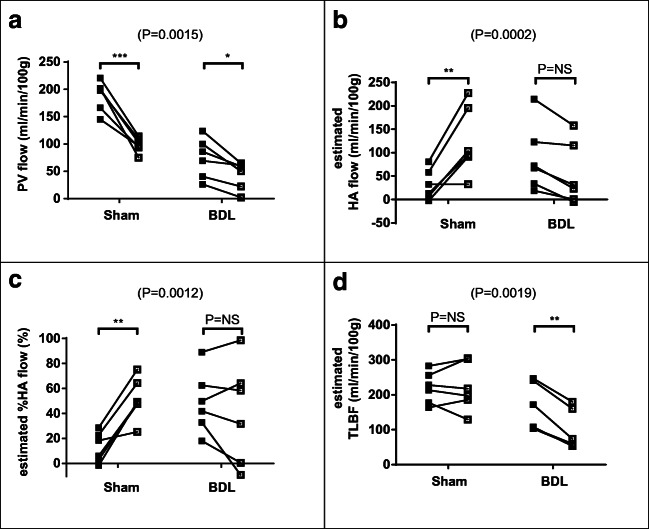
Hepatic haemodynamic parameters (**a**) PV flow, (**b**) estimated HA flow, (**c**) estimated %HA flow and (**d**) estimated TLBF at baseline (■) and following terlipressin (□) in sham-operated and BDL rats. Two-way ANOVA *p* values cited above each chart, with post hoc post-terlipressin *p* values for each cohort (*p* = NS— non-significant; *p < 0.05; **p < 0.001; ***p < 0.0001)

**Fig. 5:**
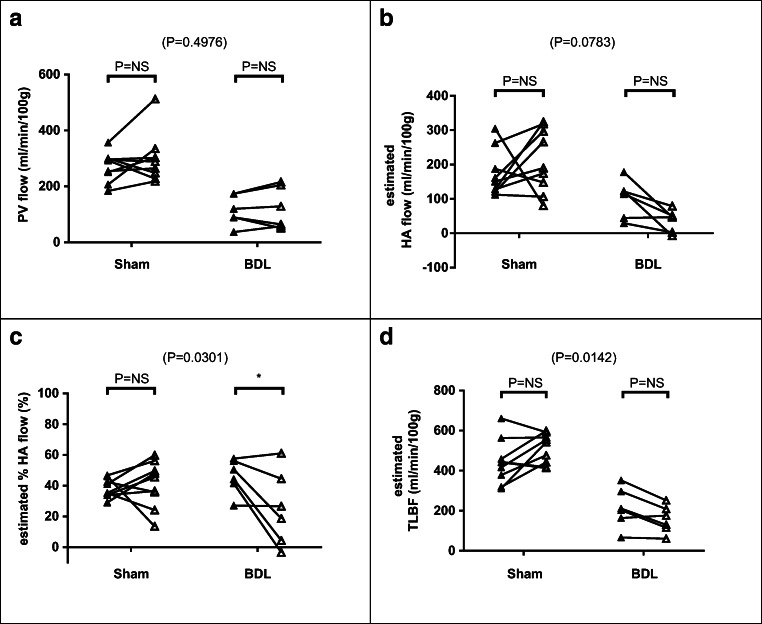
Hepatic haemodynamic parameters **a** PV flow, **b** estimated HA flow, **c** estimated %HA flow and **d** estimated TLBF at baseline (▲) and following LPS (∆) in sham-operated and BDL rats. Two-way ANOVA *p* values cited above each chart, with post hoc post-LPS *p* values for each cohort (*p* = NS—non-significant; *p < 0.05; **p < 0.001; ***p < 0.0001)

**Fig. 6:**
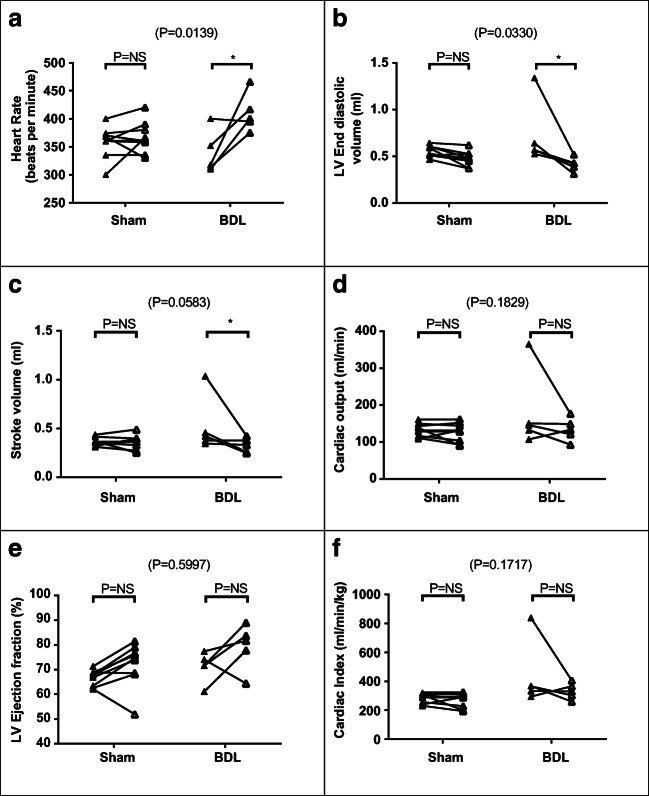
Cardiac systolic function parameters (**a**) heart rate, (**b**) LV end-diastolic volume, (**c**) stroke volume, (**d**) cardiac output, (**e**) LV ejection fraction and (**f**) cardiac index at baseline (▲) and following LPS (∆) in sham-operated and BDL rats. Two-way ANOVA *p* values cited above each chart, with post hoc post-LPS *p* values for each cohort (*p* = NS—non-significant; *p < 0.05; **p < 0.001; ***p < 0.0001)

An example of a cardiac cine MRI study is shown in Fig. 3. Baseline cardiac systolic function did not differ significantly for heart rate (sham 333 ± 8 vs BDL 341 ± 9 bpm, *p* = 0.53), but end-diastolic volume (sham 0.66 ± 0.02 vs BDL 0.77 ± 0.03 ml, *p* = 0.02), stroke volume (sham 0.42 ± 0.02 vs BDL 0.57 ± 0.04 ml, *p* = 0.006) and cardiac output (sham 140.0 ± 8.0 vs BDL 195.5 ± 15.2 ml/min, *p* = 0.007) were significantly higher in BDL than sham-operated animals. LV ejection fraction (sham 63.2 ± 2.4 vs BDL 73.7 ± 2.8%, *p* = 0.01) and cardiac index (sham 291.5 ± 13.3 vs BDL 456.1 ± 33.4 ml/min/kg, *p* = 0.0009) were also significantly higher in BDL versus sham rats. LV mass did not differ significantly between BDL and sham-operated animals (sham 0.90 ± 0.06 vs BDL 0.98 ± 0.06 g, *p* = 0.34), but LV mass index was significantly higher in BDL rats (sham 1.87 ± 0.13 vs BDL 2.30 ± 0.13 g/kg, *p* = 0.03) (Figs. 4, 5, and 6).

### Terlipressin response studies

Post-terlipressin PCMRI flow measurements were acquired on average 15.4 ± 1.3 min after infusion was commenced. Two-way ANOVA demonstrated statistically significant interactions between cohort and effects of terlipressin for all hepatic haemodynamic parameters (Table 3). Significant reductions in PV flow were observed in both cohorts (sham *p* < 0.0001; BDL *p* = 0.02; Fig. 4a). HA flow and HA fraction increased significantly in sham animals (*p* = 0.0003 and *p* = 0.0008 respectively) but were non-significantly reduced for BDL animals (*p* = 0.11 and *p* = 0.58; Fig. 4b and c). Post-terlipressin TLBF was maintained in sham animals (*p* > 0.99) but significantly reduced for BDL animals (*p* = 0.0004; Fig. 4d).

**Table 3:** Numerical change in sham and BDL hepatic haemodynamic parameters from baseline in response to terlipressin

	Sham (*n* = 6)		BDL (*n* = 6)		Two-way ANOVA	
		*p* value†		*p* value†	*F*(1,10)	*p* value
PV flow (ml/min/100 g)	− 90.3 ± 11.1	< 0.0001***	− 31.0 ± 8.0	0.0187*	18.7	0.0015*
HA flow (ml/min/100 g)	92.8 ± 21.3	0.0003**	− 34.4 ± 7.5	0.1128	31.8	0.0002**
HA fraction (%)	38.8 ± 6.5	0.0008**	− 8.3 ± 8.3	0.5802	19.9	0.0012*
TLBF (ml/min/100 g)	2.5 ± 14.0	> 0.9999	− 65.5 ± 8.5	0.0004**	17.3	0.0019*

^†^*p* value for post hoc baseline vs post-terlipressin

*p < 0.05; **p < 0.001; ***p < 0.0001

### Acute-on-chronic liver failure studies

Post-LPS PCMRI and subsequent cardiac cine MRI measurements were acquired on average 28.9 ± 2.5 min after LPS infusion was completed. Premature demise resulted in complete post-LPS PCMRI measurements being collected in *n* = 6 and post-LPS cardiac cine MRI in *n* = 5 BDL subjects.

Two-way ANOVA demonstrated statistically significant interactions between cohort and effects of LPS on hepatic haemodynamic parameters only for HA fraction (*F*(1,13) = 5.93, *p* = 0.03) and TLBF (*F*(1,13) = 8.02, *p* = 0.01; Table 4). The interaction between animal type and effects of LPS on HA flow approached significance (*F*(1,13) = 3.65, *p* = 0.08), but post hoc HA flow increases in the sham and reductions in the BDL groups (sham *p* = 0.56 vs BDL *p* = 0.29; Fig. 5b) were both non-significant. The reduction in HA fraction for BDL rats was significant (*p* = 0.03; Fig. 5c). Post hoc TLBF increased in sham animals and reduced in BDL animals (sham + 67.8 ± 28.2 ml/min/100 g, *p* = 0.06 vs BDL − 58.6 ± 34.6 ml/min/100 g, *p* = 0.23; Fig. 5d) but was not significant.

**Table 4:** Numerical change in sham and BDL hepatic haemodynamic and cardiac parameters from baseline in response to LPS

	Sham		BDL		Two-way ANOVA	
		*p* value†		*p* value†	*F*	*p* value
Hepatic haemodynamic parameters						
*n*	9	-	6	-	(1,13)	-
PV flow (ml/min/100 g)	29.5 ± 20.5	0.3594	6.44 ± 25.1	> 0.9999	0.487	0.4976
HA flow (ml/min/100 g)	38.8 ± 34.3	0.5588	− 64.99 ± 42.05	0.2924	3.652	0.0783
HA fraction (%)	2.9 ± 6.1	> 0.9999	− 20.9 ± 7.5	0.0322*	5.928	0.0301*
TLBF (ml/min/100 g)	67.8 ± 28.2	0.0639	− 58.6 ± 34.6	0.2283	8.015	0.0142*
Cardiac systolic function parameters						
*n*	9	-	5	-	(1,12)	-
Heart rate (bpm)	8 ± 13	> 0.9999	72 ± 18	0.0032*	8.278	0.0139*
End-diastolic volume (ml)	− 0.07 ± 0.06	0.4815	− 0.31 ± 0.08	0.0040*	5.894	0.0330*
Stroke volume (ml)	− 0.02 ± 0.05	> 0.9999	− 0.20 ± 0.07	0.0258*	4.378	0.0583
Cardiac output (ml/min)	− 5.5 ± 17.0	> 0.9999	− 45.6 ± 22.7	0.1358	1.999	0.1829
LV ejection fraction (%)	5.6 ± 2.9	0.1510	8.1 ± 3.8	0.1106	0.2906	0.5997
Cardiac index (ml/min/kg)	− 12.0 ± 39.2	> 0.9999	− 107.4 ± 52.6	0.1275	2.113	0.1717

^†^*p* value for post hoc baseline vs post-LPS

*p < 0.05; **p < 0.001; ***p < 0.0001

Interactions between cohort and effects of LPS on cardiac systolic function parameters were significant only for heart rate (*F*(1,12) = 8.28, *p* = 0.01) and end-diastolic volume (*F*(1,12) = 5.89, *p* = 0.03, Table 4; Fig. 6). Post hoc tests demonstrated significant post-LPS BDL rat increases in heart rate (*p* = 0.003; Fig. 6a) and reductions in end-diastolic volume (*p* = 0.004; Fig. 6b) and stroke volume (*p* = 0.03; Fig. 6c). Reductions in cardiac output were observed in both groups, but not statistically significant (Fig. 6d).

## Discussion

We applied caval subtraction PCMRI and cardiac cine MRI to a rodent model of chronic liver disease to non-invasively demonstrate significant differences in hepatic haemodynamic and cardiac systolic function parameters at baseline, following terlipressin and in ACLF. A major strength of this work is that these data were collected non-invasively in small animals for the first time. The measurement of HA flow (and TLBF) response and the haemodynamic effects of ACLF are particularly novel.

We observed reduced PV flow and TLBF, despite elevated HA flow/fraction at baseline in BDL rats. This suggests long-standing PV flow reductions are buffered by rises in HA flow, but that this response is inadequate in cirrhosis, with TLBF reduced overall. Reassuringly, our results concur with invasive hepatic haemodynamic parameters reported previously, where reduced PV flow has been demonstrated in BDL and carbon tetrachloride models of chronic liver disease[22–24] and elevated HA flow demonstrated in BDL rats [23, 25].

Elevated LV end-diastolic volume, stroke volume, cardiac output, LV ejection fraction, cardiac index and LV mass index all suggest increased baseline systolic function in BDL rats. Increased cardiac output, cardiac index and LV mass index have been reported previously in BDL rats [26–28] and elevated systolic function is a recognised feature of cirrhosis, and has been reported previously in patients with chronic liver disease [29, 30]. The precise mechanisms are not fully understood, but a proposed explanation includes the hyperdynamic circulation of cirrhosis and peripheral vasodilatation, resulting in effective hypovolaemia and arterial hypotension, which in turn drives ionotropic compensation through increased sympathetic nervous system activity [31].

Following terlipressin, we observed expected reductions in PV flow in both cohorts. In the sham group, this was buffered by increased HA flow to maintain overall TLBF, but in the BDL rats, the HA buffer response failed, with a reduction in overall TLBF. The HA buffer response has been previously demonstrated invasively in preclinical models [32], but without terlipressin or in a small animal model of cirrhosis. Terlipressin in the setting of acute variceal haemorrhage and hepatorenal syndrome is proven clinically, but the failure of the HA buffer response and sustained hepatic hypoperfusion in chronic liver disease observed in the study raises questions regarding the potential impact of prolonged use in either of these clinical settings, or indeed effects on perfusion to other critical organs such as the brain, where altered perfusion has been described previously [33].

Endotoxin-mediated ACLF induced reductions in HA fraction and differences in TLBF response overall in BDL rats. These changes occurred on a background of increased heart rate and a reduction in end-diastolic volume, but with otherwise relatively stable cardiac systolic function parameters, including cardiac output. This would suggest cardiac/systemic factors alone do not account fully for deleterious changes in HA flow in ACLF. It is also possible that the small sample size may have masked significant changes in other parameters—reductions in cardiac output and invasive indocyanine green (ICG)-determined TLBF have been demonstrated in patients with ACLF, relative to those with stable cirrhosis [13]. However, these changes have not been evaluated previously in small animals, as demonstrated here for the first time.

Our study has important limitations. Although caval subtraction PCMRI has been validated previously [15], it relies on bulk flow measurements from several vessels, so that measurement errors from each are propagated when HA flow is calculated. This can result in non-physiological negative estimations of HA flow, recorded for some BDL subjects after terlipressin and LPS.

Acquisition of a full caval subtraction PCMRI dataset took just under 30 min, so that accuracy would depend on stability of each haemodynamic parameter during that time period. For terlipressin studies, the dosage protocol was based on delivering a sustained reduction to PV flow over the measurement period; however, for LPS-induced acute sepsis, the haemodynamic effects are likely to be less predictable, with probable fluctuations in hepatic and cardiac response.

An important method to control for changes in systemic factors would be via simultaneous monitoring of mean arterial pressure, but this was impossible because of the need for additional monitoring equipment within the scanner. Terlipressin dose was similar to those used previously in rodent studies [34–36] albeit substantially higher than those used clinically. Intravenous dosing regimes for acute variceal bleeds are around 0.5 mg/h (or 0.12 μg/kg/min in a 70 kg adult), compared with 10 μg/kg/min used in this study—the reason for this may well reflect the pharmacokinetic/pharmacodynamic differences between species. Finally, LPS can trigger progressive organ failure and demise of BDL rats [37]; four subjects failed to complete our protocol. This introduces selection bias, as recorded measurements reflect only those BDL rats whose hepatic haemodynamic and cardiac reserve permitted survival.

There are several opportunities for future work. Terlipressin was used to evaluate the effects on TLBF and HA flow to a known, clinically translatable modulator of PV flow, but other vasoactive therapies administered routinely to patients with chronic liver disease that have potentially deleterious effects on TLBF—such as beta-blockers—could be investigated. The evolution of vascular complications of chronic liver disease using sequential non-invasive MRI studies in the same animal as cirrhosis/portal hypertension evolves over 4 to 5 weeks would also be interesting. Diastolic dysfunction is known to occur in chronic liver disease, and could be evaluated using mitral valvular PCMRI studies. Insights regarding diastolic dysfunction severity and response to vasoactive drugs and sepsis would be meaningful. Finally, important mechanistic questions around dysregulation of HA flow/changes in cardiac function that are associated with chronic liver disease, particularly in the setting of ACLF, are raised by this study. Further mechanistic studies evaluating systemic mediators and tissue-based factors may clarify the aetiology of poor outcomes in these patients and help identify/evaluate much needed potential vasoactive therapies.

In summary, we have used caval subtraction PCMRI and cardiac cine MRI to demonstrate reduced PV flow, increased HA flow but reduced overall TLBF, alongside elevated cardiac systolic function at baseline in a rodent model of chronic liver disease. We have demonstrated failure of the HA buffer response in cirrhotic BDL rats in response to terlipressin and an altered hepatic haemodynamic response in ACLF, with reductions in HA fraction and TLBF, despite relatively preserved cardiac systolic function. Taken together, these findings suggest that dysregulation of HA flow/fraction is a feature chronic liver disease and is perturbed further by vasoconstrictor therapies and during ACLF. Our study also emphasises caval subtraction PCMRI with cardiac cine MRI as potentially useful tools for preclinical development of new vasoactive therapies for patients with chronic liver disease.

## Abbreviations

ACLF: Acute-on-chronic liver failure
ANOVA: Analysis of variance
BDL: Bile duct ligated
HA: Hepatic artery/arterial
LPS: Lipopolysaccharide
LV: Left ventricle
PCMRI: Phase-contrast MRI
PV: Portal vein/venous
TLBF: Total liver blood flow
